# Enhancing Solar Cell Efficiency Using Photon Upconversion Materials

**DOI:** 10.3390/nano5041782

**Published:** 2015-10-27

**Authors:** Yunfei Shang, Shuwei Hao, Chunhui Yang, Guanying Chen

**Affiliations:** 1 School of Chemical Engineering and Technology, Harbin Institute of Technology, Harbin 150001, China; E-Mails: shangyunfei@hit.edu.cn (Y.S.); haosw@hit.edu.cn (S.H.); 2 Harbin Huigong Technology Co., Ltd., Harbin 150001, China; 3 Institute for Lasers, Photonics, and Biophotonics, University at Buffalo, State University of New York, Buffalo, NY 14260, USA

**Keywords:** photovoltaic, upconversion, efficiency

## Abstract

Photovoltaic cells are able to convert sunlight into electricity, providing enough of the most abundant and cleanest energy to cover our energy needs. However, the efficiency of current photovoltaics is significantly impeded by the transmission loss of sub-band-gap photons. Photon upconversion is a promising route to circumvent this problem by converting these transmitted sub-band-gap photons into above-band-gap light, where solar cells typically have high quantum efficiency. Here, we summarize recent progress on varying types of efficient upconversion materials as well as their outstanding uses in a series of solar cells, including silicon solar cells (crystalline and amorphous), gallium arsenide (GaAs) solar cells, dye-sensitized solar cells, and other types of solar cells. The challenge and prospect of upconversion materials for photovoltaic applications are also discussed.

## 1. Introduction

Fossil fuels (coal, oil, and natural gas) form the major energy source for meeting current human needs [1,2,3,4], yet cause a range of serious environmental issues. Moreover, the ever-growing consumption rate outpaces their regeneration rate, endangering the exhaustion of fossil fuels on earth. Dealing with the energy crisis is an urgent need [5,6]. Among all new competing energy sources (biomass, wind, hydroelectricity, geothermal energy, and nuclear energy), solar energy is considered to be the most abundant, renewable, and environment-friendly energy form [7,8]. The total solar power that strikes the Earth’s surface is about 100,000 terawatts, which is 10,000 times more than that consumed globally [9,10]. If just 0.1% of the sunlight that reaches the Earth’s surface could be converted by photovoltaic (PV) devices, with an average conversion efficiency of 10%, the amount would be sufficient to meet our current energy demands. The achievement of PV technology in recent decades has been tremendous [11,12,13]. The high-cost per kilowatt delivered by PV stations, however, limits its competitiveness with other sources. This is caused mainly by the inferior power conversion efficiencies of PV devices. The inability to absorb infrared (IR) light (700–2500 nm), which constitutes 52% of the energy of the entire solar spectrum, forms the major energy loss mechanism of conventional solar cells. This fundamental issue is set by the sizable bandgap of light-absorbing materials in PV devices. Crystalline silicon (c-Si) photovoltaic (PV) cells are the most used among all types of solar cells on the market, representing about 90% of the world’s total PV cell production in 2008 [14]. However, even for single crystalline silicon (Si) PV cells with a rather small semiconductor band-gap (1.12 eV, corresponding to a wavelength of ~1100 nm), the transmission loss of sub-band-gap photons can still amount to about 20% of the sun’s energy irradiated onto the Earth’s surface [15]. For PV cells with a larger band-gap, such as amorphous Si (1.75 eV) solar cells, which are limited to absorb sunlight with wavelengths below 708 nm, they manifest even higher near infrared transmission losses.

**Figure 1:**
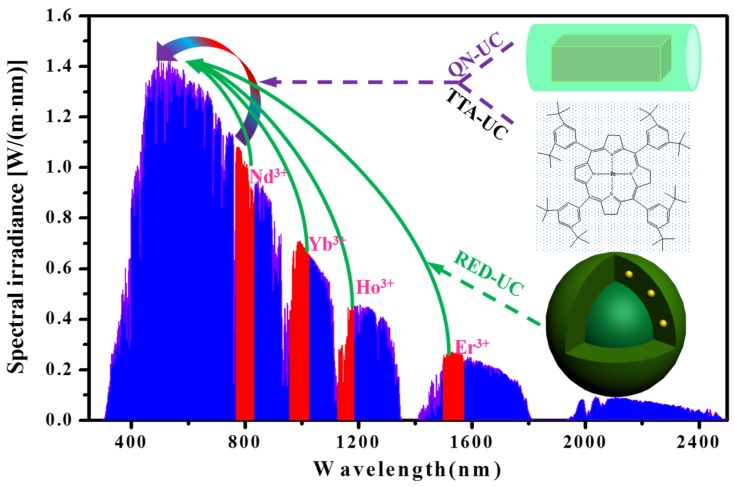
The absorption and emission range of three types of upconversion materials in reference to AM 1.5 spectrum (QN-UC (purple): upconversion in quantum nanostructures; TTA-UC (purple): triplet-triplet annihilation upconversion; RED-UC (green): Rare-earth-doped upconversion materials).

Photon upconversion (UC) provides a means to circumvent transmission loss by converting two sub-band-gap photons into one above-band-gap photon, where the PV cell has high light responsivity. This technology enables us to break the Shockley–Queisser limit of a single-junction PV cell (about 31% for non-concentrated sunlight irradiation for a semiconductor material with an optimized band-gap of around 1.35 eV) by transforming the solar spectrum [16]. Indeed, Trupke *et al.* demonstrated through a detailed balance model that modification of the solar spectrum with an up-converter could elevate the upper theoretical efficiency limit of a single-junction crystalline silicon PV cell to be as high as 40.2% under non-concentrated sunlight irradiation [17]. This value is far beyond the Shockley–Queisser limit for crystalline silicon solar cells (~1.1 eV bandgap) of approximately 30%. Figure 1 schematically illustrates the use of upconversion processes to convert the solar spectrum in the IR-Near IR (NIR)-short visible range into the peak (~500 nm) of sun radiation. There are three typical photon upconversion materials under investigation now outlined. (1) Rare-earth-doped micro- and nano-crystals (RED-UC), which usually work with wavelengths above 800 nm, but also possibly below 800 nm with appropriate composition design [18,19,20,21,22,23,24]. The abundant electronic states of trivalent lanthanide ions enable upconverting a range of IR wavelengths by selecting varied types of rare earth ions [25,26]. (2) Triplet–triplet annihilation upconversion (TTA-UC, response range λ < 800 nm) [27,28,29], whereby the triplet states of two organic molecules interact with each other, exciting one molecule to its emitting state to produce fluorescence. The excitation power density required for TTA UC is quite low, a few mW/cm^2^, which is comparable to that of sun radiation. (3) Upconversion in Quantum Nanostructures (QN-UC, response range λ < 800 nm) [30,31,32]. This process leans on the use of a unique design comprising a compound semiconductor nanocrystal, which incorporates two quantum dots with different bandgaps separated by a tunneling barrier. Upconversion occurs by excitation of an electron in the lower energy transition, followed by intra-band absorption of the hole, allowing it to cross the barrier to a higher energy state. Detailed mechanisms of these three types of upconversion materials are discussed in Section 2. These upconversion materials are now emerging in use to improve PV efficiency in the real word.

## 2. Upconversion Materials

### 2.1. Rare-Earth-Doped Upconversion Materials

The rare-earth family is comprised of 17 elements, which includes 15 lanthanide elements (from La to Lu) plus the elements of yttrium (Y) and scandium (Sc). The trivalent lanthanide ions possess a *4f^n^5s^2^5p^6^* electronic structure with 14 available orbitals (0 < *n* < 14), offering 14 possible electronic group configurations. The quantum interaction of involved electrons endows lanthanide elements with abundant energy levels covering a spectral range of NIR, visible and ultraviolet (UV) [33,34,35,36,37]. In addition, the perfect shielding of 4*f* electrons by outer complete 5*s* and 5*p* shells enables electronic transitions to occur with limited influence from the surrounding environment, thus exhibiting high resistance to processes of photobleaching and photochemical degradation. As the symmetries of involved quantum states are identical, the intra-4*f* electronic transitions of lanthanide ions are electric-dipole forbidden, yet can be relaxed due to local-crystal-field-induced intermixing of the *f* states with higher electronic configurations. The primary forbidden nature yields metastable energy levels of lanthanide ions (lifetime can be as long as tens of milliseconds), thus favoring the occurrence of sequential excitations in excited states of a single lanthanide ion as well as permitting favorable ion-ion interactions in excited states to allow energy transfers between two or more lanthanide ions.

**Figure 2:**
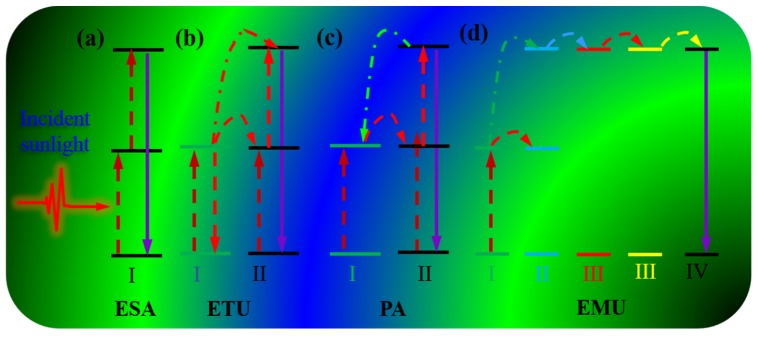
A schematic illustration of four typical upconversion processes: (**a**) Excited state absorption (ESA); (**b**) Energy transfer upconversion (ETU); (**c**) Photon avalanche (PA); (**d**) Energy migration-mediated upconversion (EMU).

There are four main basic mechanisms for rare-earth ions based upconversion processes, comprising of excited state absorption (ESA), energy transfer upconversion (ETU), photon avalanche (PA) and energy migration-mediated upconversion (EMU) (Figure 2). Excited state absorption (ESA) takes the form of successive absorption of pump photons by a single ion utilizing the ladder-like structure of a simple multi-level system as portrayed in Figure 2a. Figure 2a illustrates a simplified three level system for two sequential photon absorption processes. The ETU process involves at least two types of ions, namely a sensitizer and an activator. In this process, ion I known as the sensitizer is firstly excited from the ground state to its metastable level by absorbing a pump photon; it then successively transfers its harvested energy to the ground state and the first excited state of ion II, known as the activator, exciting ion II to its upper emitting state, which is followed by radiative decay to its ground state. The PA in Figure 2c is a looping process that involves an efficient cross relaxation mechanism between ion I in the ground state and ion II in the second excited state, resulting in generation of two ion IIs in the metastable state. The population of ion II in the second excited state is created through absorption of laser photons at its metastable state (the first excited state), which is initially populated through non-resonant weak ground state absorption. When the looping process ensues, an avalanche population of ion II will be created at its metastable state, producing avalanche upconverted luminescence from the emitting state. The generation of PA UC typically occurs above a certain threshold of excitation density. Below the threshold, very little up-converted fluorescence is produced, while the luminescence intensity increases by orders of magnitude above the pump threshold. In addition, the looping nature enables the evoked UC luminescence to be strongly dependent on the laser pump power, especially around the threshold laser power. The lanthanide ions designed for realizing energy migration-mediated upconversion (EMU) comprise four types: the sensitizers (type I), the accumulators (type II), the migrators (type III), and the activators (type IV; Figure 2d). A sensitizer ion is used to harvest excitation photons and subsequently promotes a neighboring accumulator ion to its excited states. A migrator ion extracts the excitation energy from high-lying energy states of the accumulator, followed by random energy hopping through the migrator ion sublattice and trapping of the migrating energy by an activator ion that produces luminescence by decaying to the ground state. The excitation density for RED-UC is typically in the range of 10^−1^–10^2^ W/cm^2^.

Rare-earth-doped upconversion materials typically consist of an appropriate dielectric host matrix and doped Ln^3+^ ions that are dispersed as the guest in the lattice of the host matrix. Host materials with low phonon energy are able to produce upconversion luminescence at high efficiency, as multiphonon-assisted nonradiative relaxations between the closely spaced energy levels can be minimized, thus yielding increased lifetime of intermediate energy levels. Investigated low phonon energy host materials typically include fluorides, chlorides, iodides, and bromides [38], while high phonon energy host materials such as silicates, borates, and phosphates are also under study [39]. In general, host materials with low phonon energy are hygroscopic, while the high phonon energy ones are robust even under acute environment (strong acid, base, high temperature, *etc.*). Yet, the type of fluoride host material is unique and has attracted a lot of attentions in recent years. This is because fluoride host lattice not only has low phonon energy but also shows excellent chemical stability. In particular, hexagonal NaYF_4_ lattice is considered to be one of the most efficient host materials to date [40,41,42]. Interestingly, even for cubic phase NaYF_4_, a well-defined distribution of Na^+^ and Y^3+^ ions in the crystal lattice can enable ultrahigh upconversion luminescence [43].

**Figure 3:**
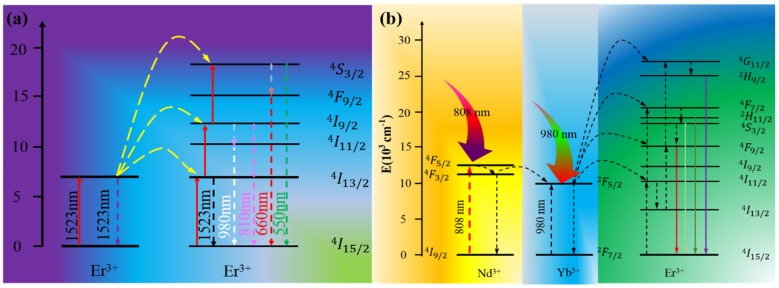
The upconversion mechanisms for (**a**) single Er^3+^ doped upconversion particles (UCNPs) excited under 1523 nm laser exciation; (**b**) Nd^3+^-Yb^3+^-Er^3+^ tridoped system under 808 nm or 980 nm laser exciation.

The Ln^3+^ dopants provide light harvesting ability as well as upconverting ability for RED UC. Among Ln^3+^ ions, the research for enhancing the efficiency of solar cells explores the use of single Ln^3+^ doping such as Er^3+^, Ho^3+^, Tm^3+^, and Pr^3+^ to upconvert IR light. Meanwhile, a utilization of Yb^3+^ ions as co-dopants can provide new, strong absorption at ~980 nm (^2^F_7/2_ → ^2^F_5/2_). The Yb^3+^ ions are able to sensitize most lanthanide activator ions, typically, Er^3+^, Ho^3+^, Tm^3+^, resulting in intense upconversion when excited. Recently, Nd^3+^ ion has been proposed as another extraordinary sensitizer. It provides a new absorption at 808 nm and is able to sensitize the Yb^3+^ ion. This further broadens the absorption range of RED upconversion for PV application. Table 1 summarizes some selected upconverting materials which can be excited, utilizing light with wavelength longer than 800 nm. (1) Single Er^3+^ doped UC materials can convert light at 1523 nm to green (550 nm) and red (650 nm) (Figure 3a) [44,45,46,47]; (2) Yb^3+^/Er^3+^ codoped system can produce green (525 nm, 542 nm), red (655 nm), as well as purple (415 nm, weak) emissions under 980 nm laser excitation [42,48,49,50,51,52,53]; (3) Yb^3+^/Tm^3+^ pairs are able to convert light at 980 nm into UV (345 nm), blue (480 nm) and NIR (800 nm) emissions [54,55,56,57,58]; (4) Nd^3+^/Yb^3+^/Ln^3+^ (Ln = Er, Tm, Ho, *etc.*) tri-doped UC materials can convert 808 nm NIR light to visible luminescence [57,58,59,60,61,62]. As an example, the energy transfer processes for Nd^3+^, Yb^3+^, and Er^3+^ are depicted in Figure 3b for illustration. The Er^3+^ ion emits in the green and red range after absorbing the excitation energy by either the Nd^3+^ or the Yb^3+^ ions. Simultaneous use of Nd^3+^, Yb^3+^ and Er^3+^ ions enables light harvesting at ~800 nm, ~980 nm, as well as ~1523 nm, covering broader spectral range for upconversion.

**Table 1:** Some selected upconverters excitable with wavelength longer than 800 nm.

Dopant Ion	Host Material	Excitation (nm)	Emission (nm)	References
Er^3+^	NaYF_4_	1523	550, 660, 800, 980	[63]
Yb^3+^-Er^3+^	NaYbF_4_	980	520, 540, 654	[64]
Yb^3+^-Er^3+^	NaYF_4_	980	410, 522, 540, 650	[65,66,67]
Yb^3+^-Er^3+^	NaYF_4_@NaYF_4_	980	510~570, 640~680	[68]
Yb^3+^-Er^3+^	NaYF_4_@NaYF_4_:Nd^3+^	808/980	520, 540, 655	[69]
Yb^3+^-Tm^3+^	NaYF_4_	980	375, 450, 475, 679, 800	[70,71]
Yb^3+^-Ho^3+^	NaYF_4_	980	545, 650	[72]
Yb^3+^-Er^3+^-Nd^3+^	NaYF_4_	808/980	410, 520, 545, 650	[60]

### 2.2. Triplet-Triplet Annihilation Upconversion Materials

**Figure 4:**
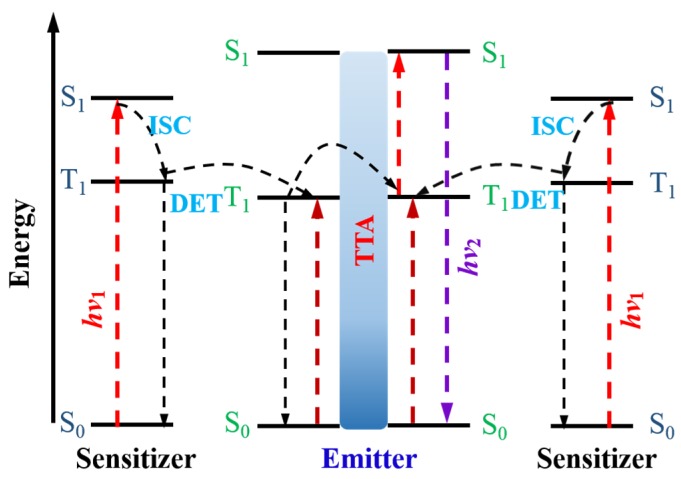
The working principle of the triplet-triplet annihilation upconversion.

Triplet-triplet annihilation (TTA) up-conversion is a process occurring in a pair of sensitizer-annihilator dyes, as portrayed in Figure 4. Light harvesting is enabled by the sensitizer, followed by populating its singlet excited state (S_0_ → S_1_). The intersystem crossing process (ISC, S_1_ → T_1_) allows the triplet excited state of the sensitizer (organo-metallic type) to be efficiently populated thanks to the heavy atom effect of the transition metal atom. Subsequently, a transfer of the energy in the triplet to a neighboring acceptor (annihilator) at S_0_ state *via* a Dexter energy transfer (DET) process, can excite the acceptor (annihilator) to its triplet state. Two nearby acceptor (annihilator) molecules in the triplet states collide with each other, and results in one acceptor molecule being excited to the higher singlet state (S_1_), while the other one returns to the ground singlet state (S_0_). A radiative decay from the generated singlet excited state of the acceptor produces an upconverted fluorescence, which is called triplet-triplet annihilation (TTA) upconversion. Furthermore, the wavelength of light harvesting can be varied by selecting the sensitizer, while the emission wavelength of TTA up-conversion can be tuned by selection of the triplet acceptor (annihilator). Since sun radiation can be utilized in a direct way to induce TTA UC, this type of upconversion is promising for applications in PV solar cells. Yet, the inability to produce efficient TTA UC in long visible as well as in the IR range, limits its impact on PV technology. To address this challenge, Kimizuka *et al.* [73] and Bardeen *et al.* [74] extended the absorption wavelength beyond 850 nm by using metallonaphthalocyanines and semiconductor nanocrystals as triplet sensitizers. However, the upconversion quantum efficiency of TTA-UC remains rather low. To address the low efficiency problem, F. Meinardi and co-workers [75] combined organic TTA with fluorescent semiconductor nanocrystals that not only broaden the absorption but also lower the excitation intensity. The same group [76] synthesized a series of sensitizers with tuned absorption and achieved a conversion efficiency of 10% under broadband AM 1.5 irradiation. Kimizuka *et al.* [77] demonstrated a novel metal-organic framework, in which donor-acceptor position could be precisely tailored to achieve a high UC efficiency even under a weak excitation (less than that of sunlight).

TTA UC is highly sensitive to oxygen molecules, as the generated triplet states of TTA dye pairs can be easily quenched by them, producing reactive oxygen species (ROS). These ROS species are exceptionally reactive, which can cause the chemical degradation of the paired dyes. As a result, TTA upconversion will be deactivated by oxygen molecules, posing another problem for their PV applications. To address this issue, Kimizuka and co-workers prepared a solvent-free liquid TTA UC system that can function well even under aerated conditions [78]. Subsequently, they reported a series of air-stable TTA upconversion in supramolecular organogel [79], supramolecular self-assemblies [80], and ionic liquids [81], providing a new approach to solve the problem of the sensitivity to oxygen molecules. Alternatively, Weder *et al.* demonstrated TTA upconversion in molecular glasses and organogels where oxygen-induced upconversion fluorescence quenching could also be prevented [82,83].

### 2.3. Upconversion in Quantum Nanostructures

**Figure 5:**
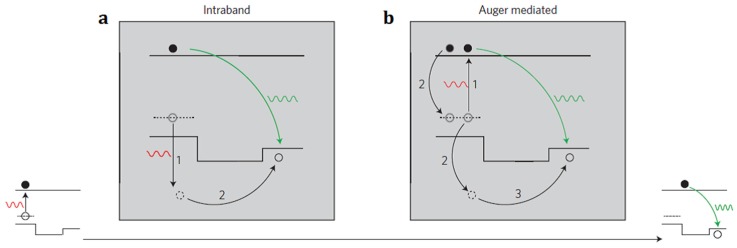
Mechanism of the upconversion in quantum nanostructures: (**a**) Direct intraband hole absorption mechanism of upconversion; (**b**) Auger-mediated upconversion (adapted with permission from [30]; Copyright Nature Publishing Group, 2013).

Upconversion in quantum nanostructures is realized through a design of a compound semiconductor nanocrystal, which incorporates two quantum dots with different bandgaps separated and connected by a tunneling barrier (a semiconductor rod). The implementation of upconversion is through a two-step absorption of two subsequent photons. The first photon generates an electron-hole pair via interband absorption in the lower-energy core (small band-gap dots), leaving a confined hole and a delocalized electron in the compound semiconductor nanocrystal. The second absorbed photon can lead, either directly or indirectly, to further excitation of the hole, enabling it to cross the barrier layer. The direct way is through an intraband absorption of the photon by the confined hole at the lower-energy core (Figure 5a), while the indirect way is via an Auger mediated energy transfer process (Figure 5b).The Auger process describes a recombination of the second electron-hole pair, generated by absorbing the second photon, while simultaneously allowing nonradiative energy transfer to the confined hole at the lower energy core (small band-gap dots), empowering it to cross the barrier to the higher energy quantum dot (large band-gap dots). This, in turn, is followed by a radiative recombination with the delocalized electron, producing upconverted luminescence. This system combines the stability of an inorganic crystalline structure, with the spectral tunability afforded by quantum confinement. Since the absorption, emission, and lifetime of the semiconductor nanocrystals can be controlled by variation of their size, shape, as well as composition, upconversion in quantum nanostructures holds prime promise for applications in PV devices. However, a relative high excitation density (~10^4^ W/cm^2^) is needed to activate this type of upconversion. Lowering the excitation density to the range of sun irradiation (~10^−1^ W/cm^2^) is an inviting direction, which could produce a pronounced impact for PV applications in a straightforward way.

## 3. PV Applications

**Figure 6:**
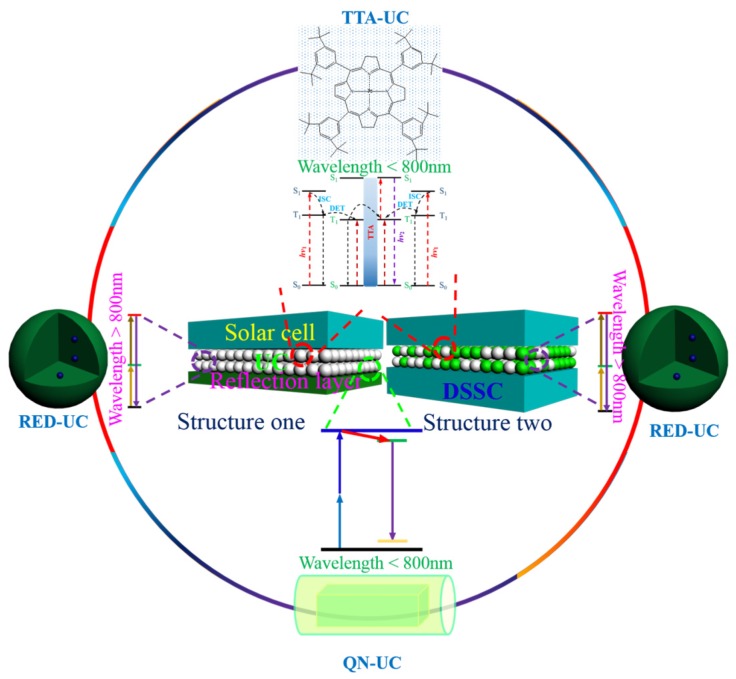
A schematic illustration of the usage of three typical upconversion materials in solar cells using two configurations. (Rare-earth upconversion, RED-UC; Upconversion in quantum nanostructure, QN-UC; Triplet-triplet annihilation upconversion, TTA-UC).

The solar cell, which directly converts solar energy into electricity, is one of the most attractive solutions to the growing energy demands, due to the omnipresence and abundance of solar energy. Light harvesting is the first but also the most important step, which determines how much sun irradiation can be absorbed. The standard solar irradiation spectrum (AM 1.5) covers the wavelength region from UV to IR (300–2500 nm). However, most single-junction PV devices are unable to absorb light quanta in the long wavelength range, typically NIR and IR, creating the performance-limiting issue of transmission loss. Upconversion materials are in play to circumvent this issue by spectral conversion, which has been applied to a wide range of solar cells. These PV devices include c-Si, amorphous Si thin-film, GaAs and dye sensitized solar cells (DSSCs). Two typical configurations have been exploited for applications in PV cells, as portrayed in Figure 6, to upconvert sub-bandgap light into above-bandgap luminescence. Structure One involves the use of a reflection layer to increase the optical path of NIR or IR light within the upconverting layer for an increase of the upconversion luminescence output, while Structure Two does not use any reflection layer, typically employed by DSSCs.

### 3.1. c-Si Solar Cells

Crystalline silicon (c-Si) photovoltaic cells are used in the largest quantity of all types of solar cells on the market, representing about 90% of the world total PV cell production in 2008. The recorded maximum conversion efficiency of crystalline silicon solar has reached 25% [84], close to the Schockley–Queisser limit (~30%). However, as mentioned before, the bandgap of crystalline silicon (~1.12 eV) limits it to absorb light less than 1100 nm, producing a transmission loss as high as 20%. On the other hand, c-Si solar cells work with the highest quantum efficiency in the spectral region of 800–1100 nm. Development of upconversion materials with absorption below 1100 nm, and the upconverted emission in the range of ~800–1100 nm are highly attractive for improvement of the efficiency of a c-Si PV cell. Yb^3+^-sensitized or Nd^3+^-sensitized RED UC materials are beyond consideration for use in c-Si PV cells, as they provide absorption at ~980 nm and ~800 nm where the semiconductor silicon has existing high spectral responses.

Single Er^3+^-doped RED UC materials are attractive for c-Si solar cells, as they display absorption at 145–1580 nm with sub-bandgap energy of silicon, and emit strong luminescence at ~980 nm, ~540 nm, and 650 nm that can be very useful for silicon to produce excitons. In 2005, Shalav *et al.* [63] exploited the use of NaYF_4_:20% Er^3+^ microcrystals as an upconverter in bifacial c-Si solar cells. In their geometric arrangement of implementation of upconversion, microsized NaYF_4_:20% Er^3+^ phosphors were dispersed into an acrylic adhesive medium with matched refractive index, and then this mixture was deposited as a thick film on the rear of a bifacial c-Si solar cell. An impressive increase of external quantum efficiency of 2.5% was accomplished under excitation at 1523 nm. Fischer *et al.* [47] also investigated the potential use of microsized or bulk NaYF_4_:20% Er^3+^ materials (~3 μm) to improve the conversion performance of c-Si solar cells. The upconversion efficiency of NaYF_4_:20% Er^3+^ microcrystals was quantified to be 5.1% when irradiated with 1523 nm laser with a power density of 1880 W/m^2^. Shalav *et al.* showed that a c-Si solar cell device can have an external quantum efficiency of 0.34% under sub-bandgap irradiation at 1522 nm with a power density of 1090 W/m^2^.

**Figure 7:**
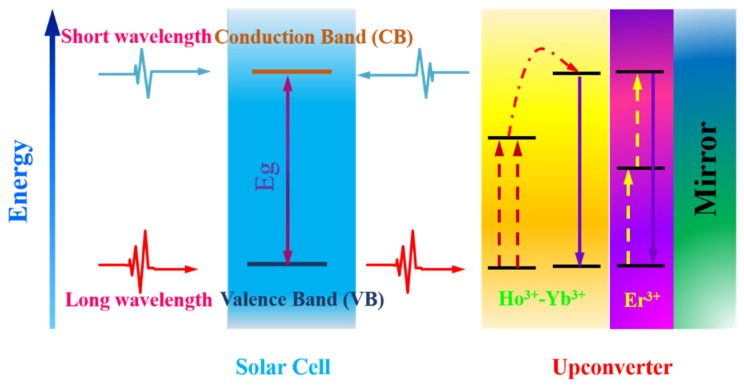
An operating mechanism for a c-Si solar cell with two upconverters, one co-doped with Ho^3+^–Yb^3+^ and the other one single doped with Er^3+^. Photons with short wavelength can be absorbed directly by the solar cell. The transmitted sub-bandgap light can be upconverted into high-energy photons, which would be absorbed by c-Si. The mirror behind the upconverter increases the probability of absorption of sub-bandgap light in the upconverter layer.

Alongside Er^3+^ ions, the Ho^3+^ ions provide a possibility to harvest sub-bandgap energy of silicon at a new wavelength range (1150–1230 nm). The centroids of upconverted luminescence of Ho^3+^ ions are at ~910 nm (corresponding to the ^5^I_5_ → ^5^I_8_ transition) in the NIR range, and at ~650 nm (corresponding to the ^5^F_5_ → ^5^I_8_ transition) in the visible range. Both radiations have energy above the bandgap of silicon. It should be noted that the intensity of sun radiation at 1170 nm is approximately twice than that at 1520 nm, potentially delivering more effective improvement of solar cell efficiency. Lahoz *et al.* [85] first reported on the use of single Ho^3+^ ion doped upconverting glass ceramics in a c-Si solar cell. They successfully demonstrated that the c-Si solar cell responds to light irradiation at ~1170 nm due to the upconverted visible emission (650 nm) and NIR emission (910 nm) in the glass ceramics. In another work of theirs, Ho^3+^-Yb^3+^ codoped upconversion materials were investigated to improve the conversion efficiency of a c-Si solar cell [86]. They found that the Ho^3+^-Yb^3+^ codoped upconverter produced much stronger NIR emission intensity than that of the single Ho^3+^-doped counterpart. This is because the excited Ho^3+^ ions (at the ^5^I_5_ state) can sensitize Yb^3+^ ions (at the ground state ^2^F_7/2_ state), making both Ho^3+^ and Yb^3+^ luminescence. This, in turn, result in an improved NIR response of Si solar cells in comparison to the one using single Ho^3+^-doped glass ceramics. Moreover, Lahoz and co-workers [86] validated the combined use of single-Er^3+^ doped RED UC material and single Ho^3+^ doped oxyfluoride RED UC material to encompass broader sunlight harvesting in the NIR range (Figure 7). This concept was demonstrated by placing the Er^3+^ doped RED UC layer to the back of the Ho^3+^ doped RED UC layer, which is then attached to the back of a c-Si solar cell. To enhance NIR light harvesting, a mirror was placed at the bottom of the cell to reflect the unabsorbed sub-bandgap sunlight back to the upconverting layers.

**Figure 8:**
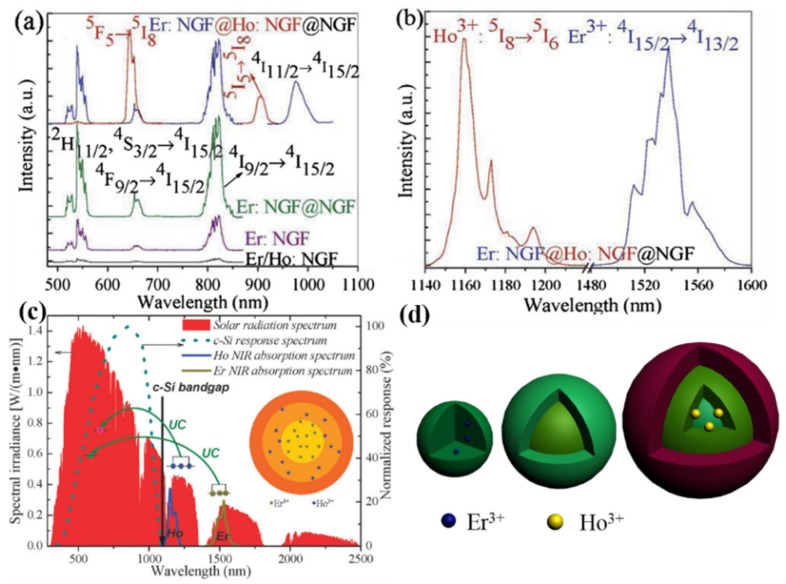
(**a**) Upconversion emission spectra of the core NaGdF_4_:Er^3+^/Ho^3+^ nanocrystals, the core NaGdF_4_:Er^3+^ nanocrystals, the core/shell NaGdF_4_:Er^3+^@NaGdF_4_ nanocrystals, and the NaGdF_4_:Er^3+^@NaGdF_4_:Ho^3+^@NaGdF_4_ core/shell/shell nanocrystals; (**b**) Upconversion excitation spectrum of the NaGdF_4_:Er^3+^@NaGdF_4_:Ho^3+^@NaGdF_4_ core/shell/shell nanocrystals; (**c**) A schematic illustration of the upconversion luminescence of NaGdF_4_:Er^3+^@NaGdF_4_:Ho^3+^@NaGdF_4_ nanocrystals *versus* the bandgap of c-for Si solar cell, and the spectrum of AM 1.5 sun irradiation; (**d**) A schematic of illustration of different nanostructures: the NaGdF_4_:Er^3+^ core, the NaGdF_4_:Er^3+^@NaGdF_4_ core/shell, and the NaGdF_4_:Er^3+^@NaGdF_4_:Ho^3+^@NaGdF_4_ core/shell/shell (adapted with permission from [87]; Copyright Royal Society of Chemistry, 2012).

From a material development point of view, Chen *et al.* [87] prepared a core-shell-shell structure of NaGdF_4_:Er^3+^@NaGdF_4_:Ho^3+^@NaGdF_4_ nanocrystals, in which the Er^3+^ and the Ho^3+^ ions were separately doped into the core and the first shell layer (Figure 8). Intense UC emissions from both Er^3+^ and Ho^3+^ were shown in the same core-shell-shell nanoparticle. The intensities of luminescence bands from both ions are all much stronger than that from Er^3+^/Ho^3+^-codoped nanocrystals due to a spatial isolation of Er^3+^ and Ho^3+^ ions, which can avoid the detrimental cross relaxation between these ions. However, because of high doping concentration of Er^3+^ and Ho^3+^, adverse relaxations could still induce quenching beyond the interfaces, even though the dopants were in different layers. To circumvent this, a multi-layer core/shell design of NaYF_4_:10%Er^3+^@NaYF_4_@NaYF_4_:10% Ho^3+^@NaYF_4_@NaYF_4_:1% Tm^3+^@NaYF_4_ nanoparticles were reported by us [88], in which the inert NaYF_4_ layers in between upconverting domains were utilized to efficiently suppress the detrimental cross-relaxation processes between different types of lanthanide ions, yielding about two times more efficient upconversion photoluminescence than the counterpart NaYF_4_:10% Er^3+^@NaYF_4_:10% Ho^3+^@NaYF_4_:1% Tm^3+^@NaYF_4_ without the inert NaYF_4_ layers. Moreover, these core/multishell nanoparticles can be excited at ~1120–1190 nm (due to Ho^3+^), ~1190–1260 nm (due to Tm^3+^), and ~1450–1580 nm (due to Er^3+^), collectively covering a broad spectral range of ~270 nm in the infrared range that can be very useful for infrared photosensitization of c-Si solar cells.

In addition, Alexander Dobrovolsky and co-workers [89] designed a system based on GaNP nanowires that can harvest IR light through a two-step two-photon absorption process and emits visible light. Though it is claimed to be suitable for application in third-generation Si-based solar cells, no demonstrations have yet been shown.

### 3.2. Amorphous Silicon Solar Cells

Amorphous Si solar cells have been considered as a promising substitute of c-Si cells due to their low cost, easy preparation, and excellent chemical stability. The band-gap of amorphous Si is larger than that of c-Si at about 1.75 eV, which confines it to absorb NIR light shorter than 700 nm. This means that upconverting materials with absorption above 700 nm should be appealing for uses in amorphous Si solar cells to circumvent the transmission loss.

Table 2 lists a range of investigated and selected single/co-doped RED UC materials in the literature that can be utilized towards this purpose. Zhang *et al.* [90] investigated the use of NaYF_4_:18% Yb^3+^, 2% Er^3+^ nanocrystals as upconverter in an amorphous Si solar cell. This kind of upconverter shows visible emissions at around 655 nm (red), 525 nm and 540 nm (green) after absorbing light at 980 nm. They showed that the short circuit density can be increased by 6.25% (from 16 to 17 mA·cm^−2^) due to the contribution of these upconverters. In analogy, De Wild *et al.* [91,92] utilized β-NaYF_4_:Yb^3+^/Er^3+^ as upconverter to enhance the power conversion efficiency of an amorphous Si solar cell. The upconverter layer with a thickness of 200–300 μm was deposited at the rear of the amorphous Si solar cell after mixing with polymethylmethacrylate. The maximum photocurrent was increased from 2.1 to 6.2 μA, along with a manifestation of an external quantum efficiency of 0.02% at 980 nm. Yb^3+^/Er^3+^-codoped TeO_2_-PbF_2_ oxyfluoride tellurite glass [93] as well as Gd_2_(MoO_4_)_3_:Er^3+^/Yb^3+^ UC materials [94] have also been investigated in a similar way by applying them at the back of amorphous silicon cells. The broader NIR light harvesting ability, due to the effect of host matrix, was supposed to yield a significant improvement in the solar efficiency. Unfortunately, there was only a tiny improvement obtained when co-excited by AM 1.5 and 980 nm laser radiation. The underlying mechanism remains unclear, and deserves further investigations.

**Table 2:** Typical dopant ions, the main emissions, and the corresponding energy transitions for upconversion nanocrystals reported in the literature.

Dopant Ion	Host Material	Excitation (nm)	Emission (nm)	Reference
Er^3+^	NaYF_4_	1523	550, 660, 800, 980	[63]
Er^3+^	YF_3_	1490	410, 530, 550, 660, 810, 980	[95]
Er^3+^	CaF_2_	1510	410, 550, 660, 980	[96]
Er^3+^	Y_2_O_3_	1538	562, 659, 801, 987	[97]
Er^3+^	Fluoride glasses	1538	550, 660, 820, 980	[98]
Er^3+^	BaCl_2_	1535	410, 550, 660, 980	[99]
Ho^3+^	Glass ceramics containing PbF_2_ nanocrystals	1170	650, 910	[85]

In addition, Er^3+^ doped β-NaYF_4_ powders were also applied to amorphous Si solar cells by Chen and co-workers [100]. This solar cell displayed a current of 0.3 μA and 0.01 μA when irradiated with a laser at 980 nm (power density of 60 mW/cm^2^) and at 1560 nm (power density of 80 mW/cm^2^), respectively. An enhanced current of 0.54 μA in solar cells was observed when irradiated with 980 nm (60 mW/cm^2^) and 1560 nm (100 mW/cm^2^) simultaneously. This was associated with a broadened NIR response due to simultaneous absorption at both ~980 nm and ~1560 nm. UC materials with broad band NIR absorption are more effective to circumvent transmission losses. In this way, TTA upconversion is in play which exploits the strong and broad absorption of dye molecules. Recently, a TTA-upconverter was investigated in hydrogenated amorphous silicon (a-Si:H) solar cells, producing a high efficiency of 10.1% with an absorption threshold of 700 nm [84]. Cheng and co-workers carried out a great deal of research work on optimizing the design of amorphous silicon solar cells combined with TTA-UC materials [101,102,103,104,105,106,107]. For example, an amorphous silicon solar cell was shown to achieve an obvious increase of current of 2.40 × 10^−3^·mA/cm^2^, on equipping with a hybrid-emitter TTA system (namely, a filled cuvette with Ag-coated glass beads) as a back-reflecting medium. The use of TTA-UC to increase the light harvest efficiency of amorphous Si solar cells is still underway.

### 3.3. GaAs Solar Cells

Gibart *et al.* in 1995 firstly reported an application of RED UC material on a GaAs solar cell, demonstrating that it is useful to enhance the solar cell efficiency by improving the harvest of unavailable IR light [108]. In their subsequent experiment [108], a substrate-free GaAs solar cell (a band-gap of 1.43 eV) was coupled to a 100 μm thick vitroceramic upconverting materials which contained Yb^3+^ and Er^3+^ ions. The efficiency of the GaAs solar cell was increased quadratically with the power of excitation due to the nonlinear nature of the upconversion process. A power conversion efficiency as high as 2.5% was achieved under 891 nm (1.391 eV) illumination with an irradiance of 25.6 W/cm^2^. In a similar way, Lin *et al.* [109] adhered a 300 μm thick UC phosphor layer of Y_5.86_W_2_O_15_:0.05Yb^3+^, 0.09Er^3+^ to the rear of a GaAs solar cell. The maximum output power of 0.339 × 10^−6^ W was obtained with a 973 nm laser irradiation at 145.65 W/cm^2^. These results clearly indicate that GaAs solar cells can work effectively under sub-bandgap light irradiance. However, the involved light irradiances are much higher than that of sun radiance (10^−2^–10^−1^ W/cm^2^). Development of efficient UC materials under low irradiance is required.

### 3.4. Dye-Sensitized Solar Cells

**Figure 9:**
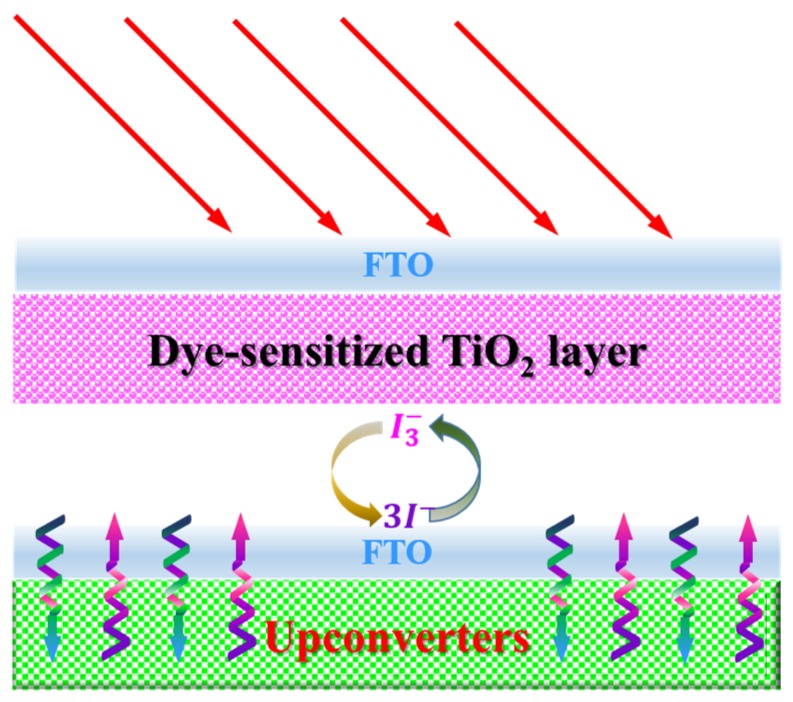
A typical schematic configuration of a DSSC equipped with upconverters.

Dye-sensitized solar cells (DSSCs) are third-generation PV cells, which have brought revolutionary innovation to PV technology since the first report by Grätzel in 1991 [110]. Owing to their low cost, simple fabrication methodology, environmental friendliness, as well as flexible structure, DSSCs have been considered as the most promising alternative for Si-based solar cells [111]. However, due to the limited absorption range of investigated dyes, the improvement of the conversion efficiency of a DSSC is always challenging [112,113,114,115]. The band-gap of typically used dyes in DSSC, such as N3, N719, and N749, *etc.*, is usually higher than 1.8 eV. This means that they are only able to absorb photons with wavelengths shorter than 700 nm, leading to unharvesting of approximately 52% of the solar energy in the IR range (from 700 to 2500 nm) [116,117,118]. Efforts to develop panchromatic sensitizers for DSSCs have been hampered by poor electron injection efficiency and competing charge recombination when the absorption spectrum of the sensitizer is extended to the IR region, adversely affecting the efficiency. Moreover, the photostability of an IR absorbing dye is known to be very poor [119,120]. Photon upconversion provides an alternative approach by converting unavailable IR photons into high-energy photons that can be absorbed by the sensitizing dyes with a typical structure as presented in Figure 9.

The first DSSC device, based on rare-earth-doped UC materials, was presented by Demopoulos *et al.*, in which LaF_3_:Yb^3+^/Er^3+^-TiO_2_ nanocomposites were used to form a multilayer electrode structure [121]. As a proof of concept demonstration, the response of DSSC at NIR light (~980 nm) was shown. Later, they deposited a layer of microsized β-NaYF_4_:Yb^3+^/Er^3+^ particles on the rear side of a counter electrode [122], which was able to provide both light reflection and NIR light harvesting without apparent charge recombination at interfaces. An improvement of NIR response was accomplished. In parallel, Yuan *et al.* [123] in 2012 proposed and validated the use of colloidal β-NaYF_4_:2% Er^3+^/20% Yb^3+^ nanoparticles in a DSSC. As the size is less than 20 nm, they are allowed to diffuse in the mesoporous TiO_2_ layer of the DSSC, enabling them to efficiently interact with sensitization dyes. The advantage of this approach is that these nanoparticles can be utilized in the way of using sensitizing dyes without modifications of device preparation procedure. Following that, Yang and co-workers attached YF_3_:Yb^3+^/Er^3+^ particles to the surface of the porous TiO_2_ thin film, the power conversion efficiency was increased from 5.18% (blank DSSC without RED UC materials) to 6.76% [124].

**Figure 10:**
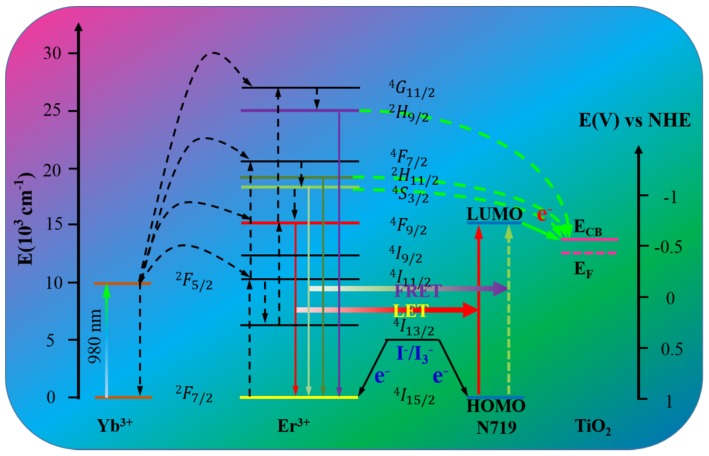
A cartoon illustration of energy transfer mechanisms in upconversion nanoparticles (UCNPs)-Doped DSSCs. This figure shows the absorption and conversion of near infrared (NIR) photons into visible light of higher energy via upconversion; the electron transfer from UCNPs to the conduction band of TiO_2_, as well as the electron transfer from I^−^/I_3_^−^ to the ground state of UCNPs. FRET and LET are fluorescence resonance energy transfer and luminescence-mediated energy transfer, respectively. HOMO and LUMO are highest occupied molecular orbital and lowest unoccupied molecular orbital, respectively.

Researchers also placed a lot of effort in the application of RED-UC structures in photoanode layers. Wang *et al.* [125] achieved an efficiency increase of 23% when doping YOF:Yb^3+^/Er^3+^ particles into the TiO_2_ photoanode layer. Zhang *et al.* exploited the use of NaYF_4_:Er^3+^/Yb^3+^@TiO_2_ core-shell composite as a photoanode. The conversion efficiency was enhanced by a factor of 1.23 [126]. To avoid electron recombination losses, Zhao *et al.* separated the upconversion and TiO_2_ layers by growing the middle SiO_2_ layer between them, achieving an improvement of 29.4% in efficiency [127]. In another way, Demopoulos *et al.* [128] investigated the utilization of β-NaYF_4_:Yb^3+^/Er^3+^@TiO_2_ submicro particles as both a light harvesting and an IR energy relay layer. An optimization of the layer of the DSSC structure resulted in a 16% relative increase of power conversion efficiency. On the other hand, there are a number of studies using a range of RED UC materials doped with Yb^3+^-Er^3+^ [68,129,130], Yb^3+^-Tm^3+^ [29], Er^3+^ [131], for improving the NIR response of DSSCs, and the following illustration (Figure 10) presents the energy transfer mechanisms in a DSSC equipped with a upconverter codoped with Yb^3+^ and Er^3+^.

Narrow absorption and high excitation threshold of RED-UC are two main limits for efficiency improvement in DSSCs. To mitigate this, Bai and co-workers [132] enhanced light harvesting by 14% due to the usage of CeO_2_:Er^3+^/Yb^3+^ nanofibers that exploited host effect to broaden the light absorption ability. Chen *et al*. [133] provided a method to broaden the NIR absorption of upconversion nanoparticles by utilization of a hexagonal core-shell structure of β-NaYbF_4_:2% Er^3+^@NaYF_4_:30% Nd^3+^. This allows simultaneous use of the absorption of Nd^3+^ (~800 nm), Yb^3+^ (~980 nm), and possibly Er^3+^ (~1550 nm) ions for light harvesting, while the core/shell structure enables a spatial isolation of Nd^3+^ from Yb^3+^ and Er^3+^ ions to avoid detrimental cross relaxations to entail a high upconversion efficiency. Zhao *et al.* [134] achieved an efficiency of 8.32% (with a noticeable enhancement of 14.78%) by putting core-shell-structured β-NaYF_4_:Yb^3+^/Er^3+^@SiO_2_@Au nanocomposites on top of a mesoporous TiO_2_ layer. Yang’s group [124,135,136] also did a lot research on enhancing the performance of DSSCs using different lanthanide ion doped nanoparticles. For example, the design of Ho^3+^-Yb^3+^-F^−^ tri-doped TiO_2_ nanoparticles enabled a 37% improvement in the power conversion efficiency. Despite recent achievements, improvement of DSSCs efficiency is still limited by the upconversion efficiency as well as the narrow and low absorption ability of RED-UC. Overcoming these two problems would lead to an epidemic use of RED-UC in DSSCs.

### 3.5. Other Types of Solar Cells

Apart from the solar cells discussed above, organic solar cells are considered to be one of the most promising alternatives for Si-based solar cells, due to their advantages of being flexible, low-cost, light-weight, of simple fabrication and large-scale production for the PV industry [137,138,139,140,141]. However, limited by the spectral mismatch of the absorption of organic molecules with sun irradiation, the improvement in efficiency remains a daunting task. Bulk heterojunction-based PV cells with a large bandgap of organic molecules are only able to absorb visible sunlight. Currently, some organic solar cells have been designed to harvest 800–900 nm sunlight by using low-band-gap polymer materials such as PCPDTBT (poly-[*N*-9ʹ-heptadecanyl-2,7-carbazole-alt-5,5(4ʹ,7ʹ-di-2-thienyl-2ʹ,1ʹ,3ʹ-benzothiadia-zole]) and its derivatives [18,142,143,144]. The poly (3-hexylthiophene) (P3HT) and the fullerenederivative [6,6]-phenyl-C61-butyric acid methyl ester (PCBM) have been exploited to increase the optical response of organic solar cells to 650–700 nm [145].

Upconversion materials have been dedicated to further extend the NIR spectral response. Wang and co-workers utilized LaF_3_:Yb^3+^/Er^3+^ phosphors to improve the NIR response of P3HT:PCBM organic solar cells. An upconverted photocurrent density of ~16.5 μA/cm^2^ was obtained under an excitation density of 25 mW/cm^2^ at 975 nm [146]. In another work of theirs, they demonstrated that the UC material (MoO_3_:Yb^3+^/Er^3+^), incorporated into P3HT:PCBM organic solar cells, contributed about 1% to the improvement of short-circuit current under one-sun (AM 1.5) illumination [147]. Wu *et al.* [148] in 2012 added NaYF_4_:Yb^3+^, Er^3+^ nanoparticles on the rear of a P3HT:PCBM organic solar cell, the short-circuit current was enhanced by 0.5 μA when illuminated by a 980 nm light.

**Figure 11:**
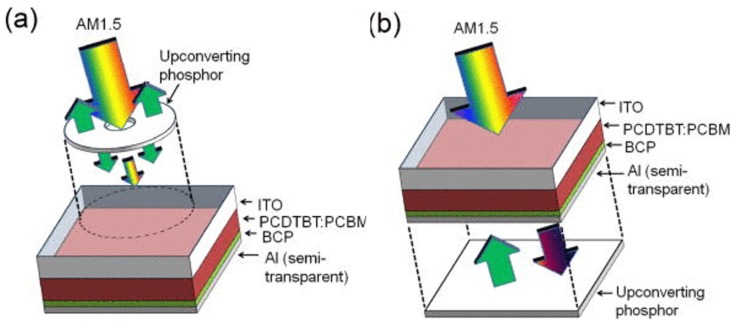
Schematic design of an organic PV device with upconversion phosphors placed (**a**) in front of or (**b**) behind the device (adapted with permission from [149]; Copyright AIP Publishing, 2012).

To utilize the NIR solar spectrum more efficiently, Adikaari *et al.* [149] reported an application of Y_2_BaZnO_5_:Yb^3+^, Ho^3+^ UC particles in PCDTBT:PCBM organic solar cells. Two different layout designs are presented in Figure 11. The PCDTBT:PCBM active layer absorbs photons of wavelength shorter than 700 nm, while Y_2_BaZnO_5_:Yb^3+^/Ho^3+^ shows an intense absorption in the NIR region of 870–1030 nm due to the ^2^F_7/2_ → ^2^F_5/2_ (Yb^3+^) transition. Moreover, the UC emission peak at 545 nm from Ho^3+^, corresponding to the ^5^S_2_/^5^F_4_ → ^5^I_8_ transition, matches well with the absorption band of PCDTBT:PCBM active layer. Illuminated by a 986 nm laser with an excitation density of ~390 mW/cm^2^, a maximum photocurrent density of 16 μA/cm^2^ and a conversion of 0.45% were obtained, showing the ability to expand the response into the NIR range. Guo *et al.* [150] used a similar method to improve the performance of both NIR harvesting and light scattering of inverted polymer BHI solar cells through mixing NaYF_4_:Yb^3+^/Er^3+^ with PCDTBT:PCBM*.*

Instead of using UC materials as upconverters, the porphyrin-based TTA-sensitizers have also been utilized to efficiently increase the NIR response of solar cells [151]. Schulze’s group carried out a comprehensive study to explore the application of TTA-UC in organic solar cells [104,106], among which the photocurrent was increased up to 0.2% under a moderate concentration of 19 suns [105]. In this system, a TTA-UC unit was conjugated to the inverted organic cell, to avoid parasitic optical losses [102].

## 4. Conclusions

In this review, we have summarized photon upconversion materials including rare-earth-doped upconversion materials, triplet-triplet annihilation upconversion materials, and quantum nanostructure upconversion materials as spectral modifiers for various types of photovoltaic solar cells to circumvent their major energy loss mechanism of transmission loss. The ability to convert the transmitted sub-band-gap photons into above-band-gap light where solar cells typically have high quantum efficiency, enables the Schockley-Queisser limit of single junction PV devices to be broken. The inorganic rare-earth-doped upconversion materials are able to work above 800 nm, while the organic triplet-triplet annihilation upconversion dye pairs typically work below 700 nm. Moreover, quantum nanostructures emerge as a new type of upconversion materials, where the size- and shape-induced quantum effects can be exploited to upconvert at therequired wavelength ranges. Indeed, the use of these upconversion materials has improved the performance of c-Si, amorphous Si, GaAs, DSSCs as well as other PV devices with varying bandgaps. Despite recent progress, the increase of efficiency remains less than 2%, far below the theoretical predication of ~10% as in the case of c-Si PV cells when irradiated with unconcentrated sun light [17]. This discrepancy is ascribed to several problems of current photon upconversion materials. (i) The established energy conversion efficiency of long wavelength rare-earth-doped upconversion materials is less than 3% [152], thus limiting the contribution of spectral conversion of absorbed photons for improvement of PV efficiency. To improve the upconversion efficiency, various approaches can be utilized such as non-luminescent impurity doping, using photonic crystals to tailor the excitation field [153], architecture of a core-shell structure to suppress surface-related quenching mechanisms, utilization of metallic structures for surface plasmon enhanced upconversion [154], *etc.*; (ii) The low and narrow absorption of rare-earth ions results in harvesting of only a small fraction of sunlight for rare earth upconversion. Design of hierarchical nanostructures to incorporate a range of rare earth ions, without introducing deleterious cross relaxations, collectively can produce intense broad band upconversion [61,155]. Alternatively, external sensitizers that have strong and broad absorption, such as organic dyes [156], quantum dots [157], and transition metal ions [158] can be utilized to sensitize rare earth ions to entail upconversion; (iii) Photon upconversion is a nonlinear optical process, defining the strong dependence of upconversion luminescence on light irradiance. Most photon upconversion materials thus have limited luminescent upconverting efficiency in the range of sun irradiance (~1000 W/m^2^). Triplet-triplet annihilation upconversion (TTA UC) has impressive efficacy under sun irradiation, favorable for uses in solar cells but with wide band-gap [101,159]. This is because the involved absorption is typically less than 700 nm [27,29,101,159,160,161,162]. Development of TTA UC with wavelengths in the IR range is appealing [102]; (iv) The upconversion in quantum nanostructures has been demonstrated at a visible wavelength, but not shown in the broad spectral range, in particular, the IR range under sunlight irradiance. This limits its uses in PV devices. Future works to overcome this problem are attractive. In all, development of upconversion materials with broad-band, strong, and tunable absorption as well as high upconverting efficiency is required, which will significantly boost solar cell efficiency by upconverison of transmitted sub-band-gap photons.
